# A Case of the Unveiling of Pulmonary Pseudoaneurysm Masquerading as a Lung Mass

**DOI:** 10.7759/cureus.38151

**Published:** 2023-04-26

**Authors:** Sushan Gupta, Avani Mohta, Danish Thameem

**Affiliations:** 1 Internal Medicine, Carle Foundation Hospital, Champaign, USA; 2 Critical Care, Carle Foundation Hospital, Champaign, USA

**Keywords:** pseudo aneurysm, tumor imaging, pulmonary hematoma, lung mass, pulmonary artery pseudoaneurysm

## Abstract

Pulmonary artery pseudoaneurysm (PAP) is an abnormal dilatation of the pulmonary vessels. They can mimic the appearance of lung nodules on chest X-rays and noncontrast CT imaging of the chest. We present a case of PAP masquerading as a lung mass for five years before presenting as a pulmonary hematoma. Our patient was an elderly male who presented to the emergency department with dizziness and weakness. He had been on regular follow-ups with annual noncontrast CT scans for a stable lung mass for the past five years. A contrast-enhanced chest CT scan on presentation showed a right lower lobe pseudoaneurysm ruptured into the pleural space with hemothorax, which was confirmed on subsequent chest CTA. The patient underwent an emergent right lower lobe resection and recovered uneventfully. Differentiating a PAP from a lung nodule is challenging and is often missed even by radiologists. A nodule or mass along the pulmonary arterial tree should raise suspicion and trigger further contrast-enhanced imaging, especially angiography, to confirm the diagnosis.

## Introduction

Pulmonary artery pseudoaneurysm (PAP) is an abnormal dilatation of the pulmonary vessels involving the external layers of the arterial wall (tunica media and adventitia) [1]. In contrast to a true aneurysm, PAPs are contained by weak surrounding tissue cover, increasing the risk of spontaneous rupture [2,3]. PAPs are uncommon, with the incidence varying between 5% and 11% among patients who undergo embolization for hemoptysis [2]. They are often associated with malignancy, infection, and trauma, which erode into the pulmonary artery leading to its formation [4]. Idiopathic PAPs are extremely rare [5]. They are often confused for lung nodules, even by radiologists, due to their similar appearance on noncontrast imaging [2]. We present a case of PAP masquerading as a lung mass for five years before presenting as a pulmonary hematoma.

## Case presentation

Our patient is an 82-year-old male with a medical history of type 2 diabetes mellitus, obstructive sleep apnea, chronic kidney disease, squamous cell carcinoma of scalp post-radiation, hypothyroidism, hypertension, hypercholesterolemia, post-orthotopic heart transplant 10 years ago, and was on immunosuppressive therapy. He presented to the emergency department (ED) with complaints of dizziness and weakness. A review of symptoms was negative for chest pain, shortness of breath, cough, and hemoptysis. He was a lifetime nonsmoker. Family, occupational, and travel history were noncontributory.

**Figure 1:**
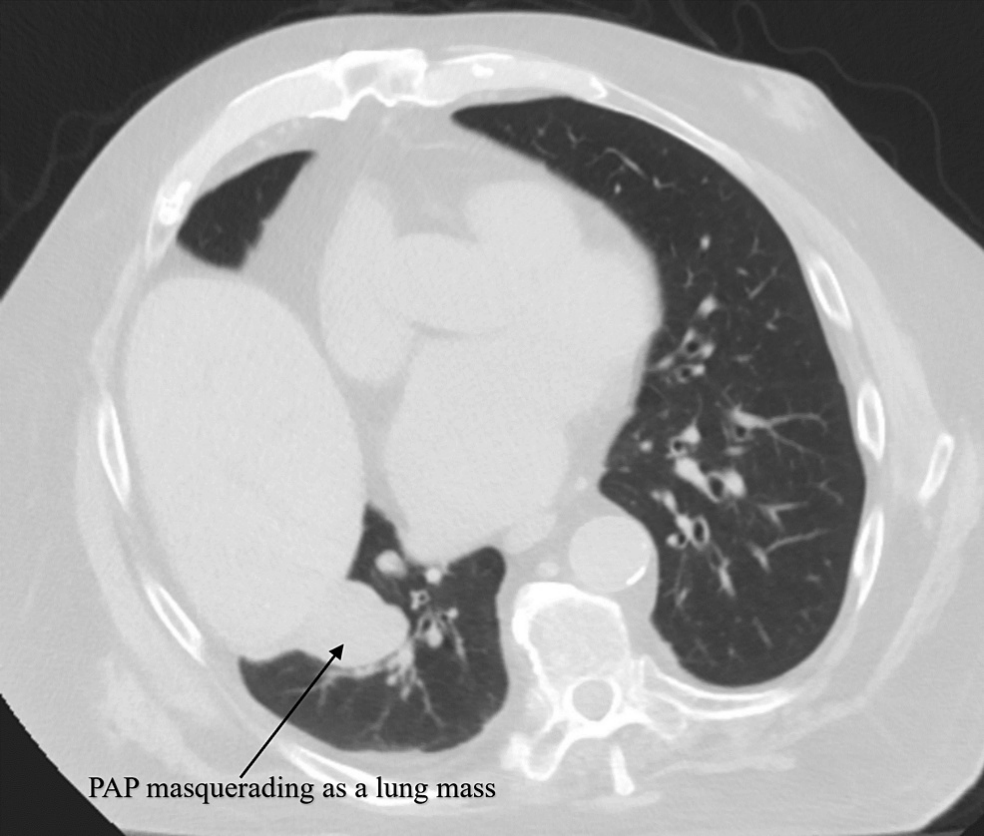
Noncontrast CT scan of lungs five years ago at the time of initial presentation PAP, pulmonary artery pseudoaneurysm

The patient had a history of a 4 cm noncalcified pulmonary mass in the posterior aspect of the right lower lobe abutting the diaphragm, which was diagnosed five years ago (Figure 1).

A PET scan at that time showed a borderline hypermetabolic mass. He refused a biopsy and has been on annual follow-ups since then. His last hospital admission was four months back, with complaints of progressively worsening shortness of breath with exertion. CT scan showed a small right pleural effusion and persistence of the right lower lobe circumscribed mass with a significantly increased size, measuring 6.0 cm in diameter. The patient’s echo showed stable cardiac function, and mild pulmonary hypertension, with a mean pulmonary artery pressure of less than 30 mmHg, unchanged from previous echocardiograms. Due to the patient's persistent symptoms, he was started on diuretic therapy every other day. He remained stable on follow-ups since then.

**Figure 2:**
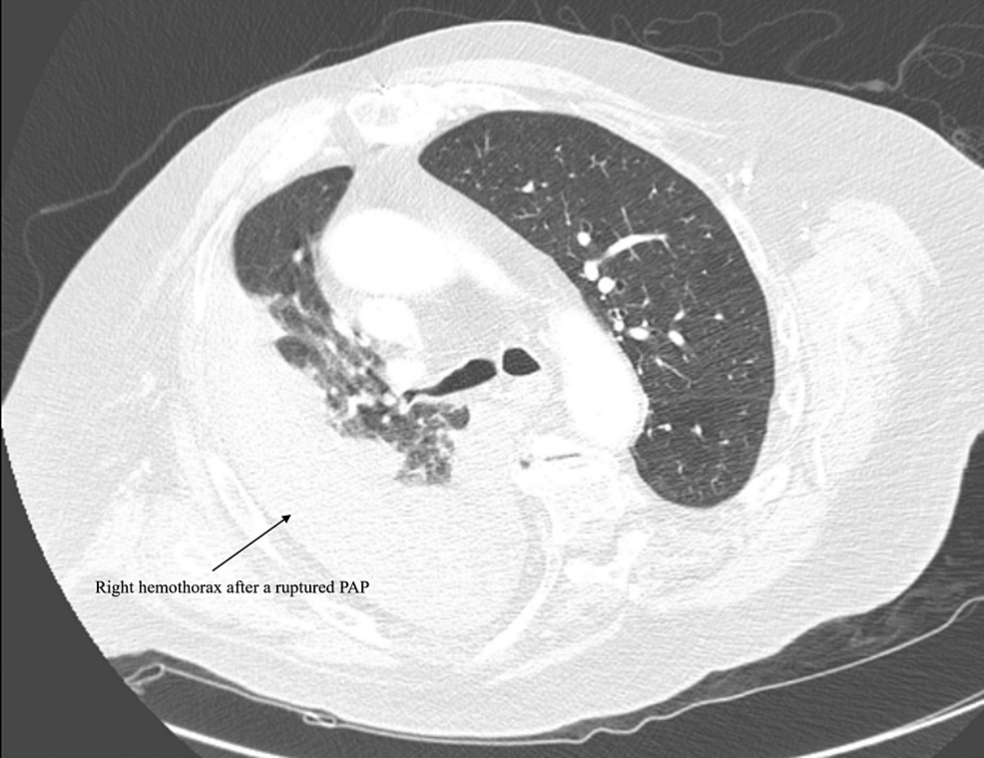
CT chest showing large hematoma in the right pleural space PAP, pulmonary artery pseudoaneurysm

On presentation to the ED during this admission, the patient was tachycardic with a heart rate of 90-100 beats/minute and hypotensive with blood pressure in the 90’s systolic. He was saturating >92% on room air. The patient received fluid resuscitation in the ED, after which the blood pressure improved. A CT chest with contrast was performed, which showed a right lower lobe pseudoaneurysm that had ruptured into the right pleural space creating a large hematoma in the pleural space (Figure 2).

**Figure 3:**
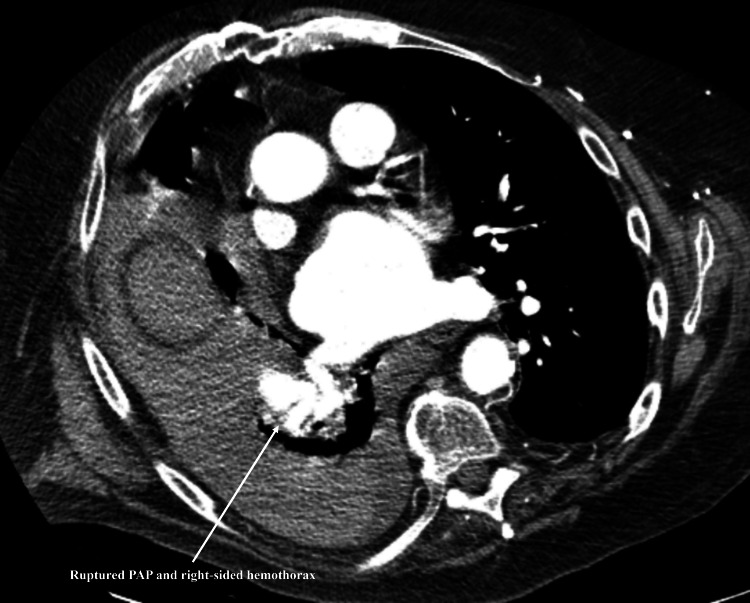
CTA chest showing right lower lobe pulmonary artery pseudoaneurysm and hemothorax PAP, pulmonary artery pseudoaneurysm

CTA showed a right lower lobe PAP with no definite acute contrast extravasation and the presence of hemothorax (Figure 3).

Cardiothoracic surgery was consulted, and the patient underwent a right thoracotomy with right lower lobe resection. He tolerated the procedure well.

Post-procedure, he was transferred to the intensive care unit for postoperative monitoring. He had an uneventful recovery. The intraoperative biopsy confirmed the ruptured PAP and associated hemothorax. At discharge, the patient was hemodynamically stable and on room air. Currently, he is on a regular pulmonary clinic follow-up with no recurrence of symptoms.

## Discussion

Pulmonary pseudoaneurysms are rare and may remain silent for years. Malignancy is often the primary differential when PAP is identified incidentally due to a similar nodular appearance on an X-ray or CT scan [1]. Physicians and radiologists often miss PAPs, with one study reporting that the radiologists missed almost half of the PAPs in their study [2]. Missed diagnosis of PAP increases the risk of life-threatening hemorrhage due to spontaneous rupture or if a biopsy is attempted to diagnose the nodule.

PAPs can either be congenital or acquired. Trauma, either iatrogenic or penetrating, is the most common acquired cause of PAP. Infections like tuberculosis, syphilis, fungal or necrotizing pneumonia, and vasculitis, especially Behcet, are other common causes [6]. Rarely, necrotizing cavitating lung malignancies may erode into pulmonary arteries forming pseudoaneurysms. On imaging, PAPs may appear as circumscribed hyperechoic nodules, making differentiation from malignancy challenging. The idiopathic presentation further adds to the diagnostic dilemma. Cough and hemoptysis may be the presenting symptoms; if associated with hemorrhage on presentation, the mortality rate is as high as 50% [6]. 

Early identification and treatment are vital. It is easy to confuse vascular structures with neoplasm due to similar hyper-echogenicity on noncontrast imaging. In rare cases, PAP can be associated with lung cancer itself, especially squamous cell carcinoma [2]. However, a nodular lesion along the pulmonary arterial tree might raise suspicion for PAP rather than a lung nodule. In a study by Chen et al. [2], most PAPs showed a predilection for segmental or subsegmental branches. Notably, even on contrast imaging, neoplasm may enhance enough to mimic the vascular structure, and vascular structure with clot may not fill up with contrast media to mimic neoplasm. Differentiating the two entities is a diagnostic challenge. CTA is the diagnostic test of choice, primarily as it identifies the precise location and size of PAP needed to plan therapy [2].

There are no specific treatment guidelines in the management of PAP. Embolization of the feeding vessel is sufficient for smaller PAPs; larger PAPs often need direct coil embolization. A surgical approach involving thoracotomy, aneurysm resection, and lobectomy is only recommended if there is associated uncontrolled hemorrhage or pleural hemorrhage, as seen with our patient [7].

## Conclusions

A PAP may mimic malignancy on noncontrast lung imaging. A nodular lesion along the pulmonary arterial tree should raise suspicion for a PAP, and CTA is often needed to differentiate the diagnosis. Early identification and treatment are essential due to the risk of life-threatening hemorrhage associated with PAP.
